# Statin-induced myopathy in a usual care setting—a prospective observational study of gender differences

**DOI:** 10.1007/s00228-016-2105-2

**Published:** 2016-08-02

**Authors:** Ilona Skilving, Mats Eriksson, Anders Rane, Marie-Louise Ovesjö

**Affiliations:** 1 Division of Clinical Pharmacology, Department of Laboratory Medicine, Karolinska University Hospital Huddinge, 141 86 Stockholm, SE Sweden; 2 Department of Endocrinology, Metabolism and Diabetes; Center for Biosciences, Department of Medicine, Karolinska Institutet, Karolinska University Hospital Huddinge, 141 86 Stockholm, SE Sweden

**Keywords:** Statin, Clinical study, Myopathy, Gender, Adverse drug reaction

## Abstract

**Purpose:**

The study aims to identify the occurrence and remission of statin-induced myopathy including patient perception and symptom characteristics with a gender perspective.

**Methods:**

The study was designed as a prospective, non-interventional investigation in 192 outpatients receiving statin treatment in usual care with 12 months follow-up. Main outcome measure was myopathy related to statin treatment and classified as probable using WHO criteria for adverse drug reaction (ADR) assessment.

**Results:**

Fourteen percent developed myopathy, risk ratio for women 1.52 [95 % CI 1.37; 1.66] as compared to men. The majority graded their pain as “severe.” CK values were within normal range. Eighty percent of the women compared to 43 % of the men reported that the muscular symptoms affected their daily life activities to a moderate or severe extent. For those who stopped treatment, mypopathy was the reason for 70 % of the women and 25 % of the men. There was a difference in mean dose between men with and without myopathy, but not in women. Among the patients with myopathy, 76 % reported other ADRs as compared to 21 % of the patients without myopathy (*p* = 0.002). Twenty-nine percent of the women and 18 % of the men reported other ADRs.

**Conclusion:**

Women reported a higher frequency of myopathy and other ADRs as well as a larger impact on daily life activities. In men, but not in women, the risk of myopathy was dose-dependent. Patients with myopathy were more susceptible to other statin-induced ADRs which raises the question about common underlying mechanisms.

**Electronic supplementary material:**

The online version of this article (doi:10.1007/s00228-016-2105-2) contains supplementary material, which is available to authorized users.

## Introduction

HMG Co-A reductase inhibitors have proven to efficiently lower serum concentration of low-density lipoprotein cholesterol (LDL-C-C) and to reduce the risk of cardiovascular disease (CVD). Since their introduction in the mid-80s, the indication for using statins in clinical practice has been well-established and few other drugs have been so widely used for such a long time [1–6]. Generic statins remain a corner stone in CVD prevention. New lipid modifying drugs such as PCSK9 inhibitors are available but are not considered cost-effective for the majority of patients [7]. The withdrawal of cerivastatin in 2001 highlighted the ability of statins to cause more or less severe myopathy including e.g., rhabdomyolysis. Today, the frequency is considered to range from 5 [8, 9] to 15 % [10, 11] depending on definition of myopathy and study design. Several studies have indicated that women seem to be more susceptible for muscular adverse drug reactions (ADRs) than men, especially elderly women. The reasons for these differences are unknown. Little attention has been paid to sex differences and myopathy [12]. In general, women were underrepresented in early large scale randomized trials such as the 4S study (19 %), the CARE study (14 %), the LIPID study (17 %), and more surprisingly also in more recent studies such as the PROVE-IT study (22 %) [13–16]. The PRIMO study and the JUPITER study included more women, 35 and 38 %, respectively [17–21]. Regardless of the reported frequency of ADRs, myopathy in routine care is a clinical dilemma and could constitute a major reason for the poor adherence to statin therapy that has been demonstrated in many studies [11, 22–24]. Statin treatment differs between women and men as was recently shown by Ballo et al. who also demonstrated that the benefits were equal if the same dose was used, but side effects was not within the scope of this study [25].

In this study, we sought to investigate the statin use and statin management in usual care with the gender disparities as main focus. We sought to identify the occurrence and remission of myopathy and include patient perception and symptom characteristics. Aware of the sensitive task to collect data on muscular side effects without interfering with the patient’s own perception, we chose a pragmatic study design, allowing both patients that were statin naïve and patients that switched the statin to be included. This design shortened our timelines allowing the study team to follow naïve subjects “into myopathy” and myopathy patients “to remission” during 12 months in usual care.

## Methods

### Study design

The study was designed as a prospective, non-interventional investigation in outpatients receiving statin treatment during a 12 month period. It encompassed 192 patients from 20 sites (primary care and cardiology outpatient clinics) in the Stockholm County. Swedish speaking patients older than 18, who in the opinion of their physician should start statin treatment or switch to another statin, were included. No intervention with the selection of patients or with statin management was made. Patients were followed for 12 months with two or three visits. Visits took place at start of the study, at 1–3 months, 6–9 and at 9–12 months thereafter. The first eligible patient was included in June 2007, and last patient visit was in December 2010.

### Ethics

This study was approved by the Regional Ethics Committee in Stockholm and the Swedish Medical Products Agency (MPA) EudraCT 2006–00,486-34. The study complied with the Declaration of Helsinki of The World Medical Association and the International Conference of Harmonization Guideline of Good Clinical Practice (ICH-GCP, E6).

### Participants and clinical procedures

All participants signed an informed consent before the start of the study. We used case report forms (paper or electronic), patient questionnaires, and standardized interviews for data capturing. Medical history included information on previous and current CVD, other diseases and life style factors e.g., smoking status, alcohol intake, and exercise. Information on concomitant medication was collected and categorized by ability to interact with CYP3A4 metabolism. Physical examination included blood pressure, waist circumference, bodyweight, and height. CVD risk classification was performed according to Reiner et al. [26]. Blood samples were analyzed for total plasma cholesterol (TPC), low density lipoprotein cholesterol (LDL-C), high density lipoprotein cholesterol (HDL-C), triglycerides (TG), apolipoprotein A1 and B, creatinine kinase (CK), myoglobulin, creatinine, hs-CRP, thyroid stimulating hormone (TSH), aspartate aminotransferase (AST), and alanine aminotransferase (ALT). Blood samples were analyzed at the Karolinska University Laboratory.

### Patient questionnaires and standardized interviews

The questionnaires covered muscular symptoms, other symptoms known to be related to statin treatment, daily life activities, statin compliance, educational level, lifestyle factors, and VAS EQ-5D (quality of life instrument) [27]. There were fixed response alternatives on muscle symptoms and their impact on daily life activities on a four-step categorical scale and on a numerical scale (NRS, 1–10). For quality of life measurement, we used EQ-5D self-rated VAS scale (graded 1–100) the two endpoints, labeled 1 = “worst imaginable health” and 100 = “best imaginable health.”

### Definition of muscular adverse drug reaction

To define myopathy, we used the criteria of the American College of Cardiology (ACC), American Heart Association (AHA), and National Heart, Lung, and Blood institute (NHLBI) [24]. The ADRs from muscles and the time to onset were assessed only in the “de novo treated” patients. In the “switch” group, we assessed if muscular symptoms improved after the statin switch. Time to onset was categorized as 0–90, 91–180, 181–270, or >270 days after start.

### ADR evaluation

The endpoint evaluation of ADRs was based on physicians’ reports, patient questionnaires, and standardized telephone interviews and reviews of patients’ medical records. An increase of two grades on the NRS and/or one step at the categorical scale was considered clinically relevant. Symptoms meeting these criteria were assessed and classified as “probable” or “possible” according to WHO, or neither “probable” nor “possible” [28].

Other ADRs such as gastrointestinal symptoms or headache are referred to as “other ADRs, only the ones assessed as ‘probable’” were included. An increase of two grades on the NRS and/or one step at the categorical scale was considered clinically relevant.

All patients who were eligible and who initiated statin treatment or made a switch were included in the full analysis set and are presented in Table 1. All patients included in the analysis reported that they took their statin 5–7 days per week. Baseline characteristics and data from primary endpoint analysis are presented for all patients who started with statin treatment and continued to first follow-up. In the analysis of myopathy, only de novo patients with a probable ADR were included.

**Table 1:** Baseline demographic and disease characteristics

Patient characteristics at baseline	Total (*N* = 180)	Women (*N* = 89)	Men (*N* = 91)	*p* ^*^
Age (years), mean (SD)	64.7 (10.4)	68.1 (8.9)	61.3 (10.6)	0.001
Age > 75 years, *n* (%)	33(18.3)	22 (24.7)	11 (12.1)	0.034
Low CVD risk, *n* (%)	8 (4.4)	3 (3.4)	5 (5.5)	0.720
Moderate CVD risk, *n* (%)	87 (48.3)	55 (61.8)	32 (35.2)	0.001
High CVD risk, *n* (%)	36 (20.0)	11 (12.4)	25 (27.5)	0.022
Very high CVD risk, *n* (%)	49 (27.2)	20 (22.5)	29 (31.9)	0.180
Muscular pain/soreness VAS score, mean (SD)	1.9 (2.2)	2.3 (2.36)	1.5 (2.0)	0.822

*Analysis for significance on continuous data level: independent t-sample test used. Levene’s test for equality of variances. Significance value two-tailed. On categorical data on ordinal or nominal level, significance was analyzed with chi-squared test/Fishers exact test, using non-parametric Levene’s test

Additional patient characteristics at baseline are shown in Supplementary Table S1

#### Statistics

Independent *t* test and Fisher’s test exact (two-sided) were used to compare factors in the myopathy group with the group without myopathy. The responses to daily life activity (DLA) were dichotomized.

All results are expressed as means ± SD, or proportion/percentages for parametric and non-parametric data, respectively. All analyses and descriptive data were performed using SPSS 22.0.

### Equipotent doses

Simvastatin was the most widely used statin in Sweden during the study period and was therefore used as a reference drug for equipotent doses (based on their ability to lower LDL): *simvastatin 5–10 mg* = pravastatin 10–20 mg = fluvastatin 20–40 mg. *Simvastatin 20 mg* = pravastatin 40 mg = atorvastatin 5 mg. *Simvastatin 40 mg* = rosuvastatin 10 mg = atorvastatin 10 mg [29].

## Results

### Patient flow and patient characteristics at study start

One hundred ninety-two individuals were screened for study participation whereof 12 subjects were assessed as screening failures. Of the remaining 180 eligible individuals, 120 were “de novo” patients and 60 “switch” patients, whereof 49 and 50 % females, respectively. Seventy-eight percent of the women completed the study and 86 % of the men. Of those who pre-terminated the study, 70 % of the women and 25 % of the men did so due to muscular symptoms.

At baseline, 35 % of the women and 58 % of the men stated no muscular symptoms. Of the patients experiencing muscular symptoms at study start (pain, weakness, soreness), 65 % of the women and 42 % of the men described “mild” or “moderate” problems with a mean pain score (NRS) 2.9 (SD 3.2) for women and 1.9 (SD2.6) as “moderate” (*p* = 0.01). No patient described their muscular status as “severe” at study start. Patient characteristics are summarized in Table 1.

### Statin treatment and myopathy in the de novo-treated patients

In all, 17 patients (14 %) of the patients experienced myopathy. Seventeen percent of the women and 12 % of the men had myopathy; all of which had myalgia. For women who started statin treatment, the risk ratio (RR) was 1.52 [95 % CI 1.37; 1.66] for myopathy compared to men. There were no cases of myositis or rhabdomyolysis. All patients had CK values within the reference range throughout the study.

Twenty-six percent of the elderly (≥75 years) had myopathy as compared to 14 % of the other patients, RR 1.84 [95 % CI 1.61; 2.06]. Given the limited number of elderly in the study, no further analysis was made on the differences between these age groups or the interaction between age and gender.

All patients with myopathy had been prescribed simvastatin, mean dose 25.3 mg (SD10.1) as compared to patients without myopathy 22.4 mg (SD 8.7), *p* = 0.235. Women in the myopathy group had a mean dose 21.0 mg (SD7.4) as compared to 22.5 mg (SD8.6) for women without myopathy, *p* = 0.626. The mean dose for men in the myopathy group was 31.4 mg (SD10.7), as compared to 23.7 mg (SD 9.4) for men without myopathy, *p* = 0.017.

All patients but one (16/17) with myopathy noticed the symptoms within the first month of statin treatment, and the 17th patient had symptoms shortly after a dose increase after 6 months of treatment.

Muscle pain increased with an average of 6.0 (SD 2.2) on NRS, in women +5.3 points (SD 2.6), in men +7.0 (SD 2.5). The intensity of myopathy was graded as mild for 12 % (2/17) (50 % women), as moderate for 35 % (6/17) (67 % women), and as severe for 53 % (9/17) (56 % women). Sixty-seven percent reported that the muscular symptoms affected their daily life activities to a “moderate or severe” extent, 80 % of the women (8/10), and 43 % (3/7) of the men (*p* = 0.001).

Patients with both muscular pain and weakness reported that their daily activities were affected to a greater extent than patients with pain only.

Myopathy was more frequent among patients at “very high CVD risk” than the other CVD risk categories (*p* = 0.048). There was no difference in other patient characteristics such as body weight or renal function, see Supplementary Table S2.

### Sub-analysis of the de novo group

Among the patients with myopathy, 76 % reported other ADRs as compared to 21 % of the patients without myopathy (*N* = 103; *p* = 0.002). These ADRs were also noted during the first follow up period. Twenty-three percent of the patients reported 38 “other ADRs”. Twenty-nine percent of the women and 18 % of the men reported at least one other ADR. There were 16 reports of sleep disturbance, 15 of gastrointestinal problems, and 5 reports of skin rash. Elevated liver enzymes were observed in two patients.

### The switch group

Among patients with muscular symptoms at study start in the “switch” group, the symptoms resolved within 3 months in 17 of 25 (68 %). These patients continued their statin treatment throughout the study. In the other patients (32 %; all women), the muscular symptoms persisted despite additional switches and/or decreased statin dose. These patients discontinued statin treatment before the study ended.

## Discussion

In our study, we examined the frequency of myopathy among women and men on statin treatment in usual care including its time to onset and remission. We found that among all subjects, 14 % experienced myopathy, 17 % of the women, and 12 % of the men. All cases of myopathy were myalgia with CK levels within normal range. This frequency of myalgia is consistent with 13 % reported by Riphagen et al. [7]. However, in that study, the assessment of the relation between myalgia and statin treatment was made by the patients. Our results represent a cautious estimate of the myopathy frequency since we included only cases assessed as at least probable.

As in several other studies, we also found that myopathy was overrepresented among women and patients older than 75 years [20, 24, 30]. We had 50 % women in our study population which reflect the gender distribution of the patients who purchased statin prescription in Stockholm County during this period. The study population did not fully reflect the proportion of elderly among statin-treated patients in the region, 18 versus 28 % (The Swedish Prescribed Drug Register). Information about the percentage of elderly in other studies is not readily available, since most studies only report mean age of the patients.

Statin myopathy is considered to be dose-dependent. We also found a significant difference in mean dose between men with and without myopathy, but between the women, there was no difference. However, 20 % of the women in the myopathy group had a concomitant medication with a potential to inhibit CYP3A4, thereby increasing the plasma concentration of some statins. Even so, these findings point to the need to better understand the mechanisms of statin-induced myalgia in women and men, respectively.

The myopathy occurred within the first month of treatment, as previously shown [20, 31]. There were no gender disparities in time to onset. However, men reported higher pain intensity, and women reported greater impact on daily life activities. The analysis also indicated that patients at very high risk for CVD were 30 % more likely to experience myopathy as compared to those with lower degrees of CVD risk. From a public health perspective, it is a challenge if those who would benefit the most from statin treatment would tolerate it the least. The unsatisfactory low adherence during long-term therapy, as has been demonstrated in several studies [32–34], may have a two-folded background. It seems likely that early discontinuation is caused by myopathy and/or other ADRs, since these symptoms usually occur within the first treatment month. For the late discontinuers, other factors such as “lack of motivation” to continue a risk modifying treatment could play a bigger role. Therefore, the time slot for assessment of long-term adherence is critical for the final estimate.

Of those already experiencing myopathy at the time of inclusion, the symptoms resolved in 68 % of the patients after switch to another statin. A similar high percentage of successful switches has been reported by Harris et al. [35]. Our sub-analysis showed that the vast majority of patients with a myopathy also reported other statin-related ADRs, which raises the question of common underlying mechanisms.

Further studies on common as well as gender specific causes of myopathy and other ADRs are warranted to enable an optimization of statin treatment to further improve the secondary prevention of CVD in women and men. Our study suggests special considerations of the gender difference, women appearing to have more side effects and larger impact on daily life activities than men.

## Electronic supplementary material

ESM 1 (DOCX 17 kb)

ESM 2 (DOCX 23 kb)
