# Supplementary material for: Statin-induced myopathy in a usual care setting—a prospective observational study of gender differences
Source: Eur J Clin Pharmacol. 2016 Aug 2;72(10):1171–6. doi: 10.1007/s00228-016-2105-2 (PMC5021730; doi:10.1007/s00228-016-2105-2)
Supplement: Supplementary file 1 — (DOCX 17 kb) [file 228_2016_2105_MOESM1_ESM.docx]

| **Additional Patient Characteristics at Baseline** | **Total (N=180)** | **Women (N=89)** | **Men**  **(N=91)** | **p^a^** |
| --- | --- | --- | --- | --- |
| Smoking, n (%) | 15 (8.3) | 9 (10.1) | 6 (10.0) | 0.431 |
| At-risk drinking^b^, n (%) | 23 (12.8) | 5 (6.1 ) | 18 (20.9) | 0.006 |
| Exercise non-regularly^c^, n (%) | 80 (44.4) | 42 (47.2) | 58 (63.7) | 0.56 |
| BMI (kg/m^2^), mean (SD) | 27.2 (4.4) | 27.0 (5.1) | 27.4 (3.6) | 0.570 |
| Waist circumference (cm), mean (SD) | 96.8 (12.8) | 94.9 (14.2) | 98.7 (11.1) | 0.057 |
| Waist circumference at risk^d^, n (%) | 90 (50.0) | 58 (65.2) | 32 (35.2) | 0.0001 |
| Diabetes, n (%) | 29 (16.1) | 13 (14.6) | 17 (18.7) | 0.550 |
| Systolic blood pressure (mmHg), mean (SD) | 139.9(19.3) | 138.7(21.7) | 139.9(16.7) | 0.676 |
| Diastolic blood pressure (mmHg), mean (SD) | 81.3 (9.9) | 80.0 (9.2) | 82.7(10.4) | 0.073 |
| EQ VAS, mean (SD) | 78.6 (14.6) | 78.0 (16.7) | 80.3 (12.6) | 0.334 |
| EQ VAS < 75, n (%) | 45 (25.0) | 26 (30.6) | 19 (24.7) | 0.483 |
| GFR^e^ (ml/min), mean (SD) | 84.6 (15.8) | 72.3 (9.1) | 96.6 (12.8) | 0.0001 |
| GFR >90 (ml/min), n (%) | 69 (38.3) | 3 (3.4) | 66 (72.5) | NA |
| GFR 60-89 (ml/min), n (%) | 104 (57.8) | 79 (89.9) | 25 (27.5) | 0.0001 |
| GFR 30-59 (ml/min), n (%) | 6 (3.4) | 6 (6.8) | - | NA |

**Supplementary Table S1. Additional Baseline Demographic and Disease Characteristics**

^a^ Analysis for significance on continuous data level: Independent t- sample test used. LevenestTest for equality of variances. significance value 2-tailed. On categorical data on ordinal or nominal level, significance was analyzed with Chi-squared test/Fishers exact test, using Non-parametric levenestest

^b^ Women > 9 glasses=108 gram alcohol per week . Men >14 glasses =168 gram alcohol per week

^c^ Exercise non-regularly= exercise less than twice weekly

^d^ Women >88 cm; Men > 102 cm

^e^ Estimated GFR according to Cockcroft-Gault
