# Supplementary material for: Statin-induced myopathy in a usual care setting—a prospective observational study of gender differences
Source: Eur J Clin Pharmacol. 2016 Aug 2;72(10):1171–6. doi: 10.1007/s00228-016-2105-2 (PMC5021730; doi:10.1007/s00228-016-2105-2)
Supplement: Supplementary file 2 — (DOCX 23 kb) [file 228_2016_2105_MOESM2_ESM.docx]

| **Characteristics of de novo tretated patients with and without myopathy [n=numbers; %=column %]** | **Patients with myopathy (N=17)** | | | **Patients without myopathy  (N=86)** | | |
| --- | --- | --- | --- | --- | --- | --- |
|  | Total | Women=10 | Men=7 | Total | Women=40 | Men=46 |
| Age (years), mean (SD) | 66.7 (12.6) | 70 (10) | 60.0(15) | 63.9 (10.2) | 68(8) | 60.7 (11) |
| Age >75 years, n (%) | 5 (29.0) | 4 (40.0) | 1 (14.3) | 14 (16.3) | 8 (20.0) | 6 (13.0) |
| Waist circumf. (cm), mean (SD) |  | 94.2 (12.6) | 102.0 (13.2) |  | 93.8 (13.7) | 95.7 (11.2) |
| BMI (kg/m^2^), mean (SD) | 26.3 (3.9) | 25.5 (4.1) | 27.3 (3.6) | 26.9 (4.5) | 27.5 (5.5) | 26.4 (3.3) |
| At-risk drinking^b^, n (%) | 2 (11.8) | 1 (10.0) | 1 (14.3) | 11 (12.8) | 3 (7.5) | 8 (17.4) |
| Excersize regularly^c^, n (%) | 10 (58.8) | 7 (70.0) | 3 (42.8) | 44 (51.2 ) | 25 (62.5) | 19 (41.3) |
| GFR ^a^ 30-59(ml/min), n (%) | 1 (5.0) | - | - | 1 (1.2) | 1(2.5) | - |
| GFR 60-89 (ml/min), n (%) | 13 (65.0) | 10 (100) | 2 (28.6) | 50 (58.1) | 38 (95.0) | 12 (26.1) |
| GFR >90 (ml/min), n (%) | 6 (30.0) | 0 | 5 (71.4) | 35 (40.7) | 1 (2.5) | 34 (73.9) |
| GFR (ml/min), mean (SD) | 81.3 (19.2) | 71.0 (10.0) | 95.9 (18.5) | 86.9 (16.1) | 73.6 (8.3) | 96.1 (14.7) |
| Interacting drugs, n(%) | 2 (11.7) | 2 (20.0) | - | 2 (2.3) | 1 (2.5) | 1 (2.2) |
| Simvastatin (mg), mean (SD) | 25.9 (10.0) N=17 | 21.0(7.4) | 31.4(10.7) * | 23.5 (8.6) N=61 | 23.0 (7.3) | 23.7(9.4)* |
| Rosuvastatin (mg), mean (SD) | - | - | -- | 16.2 (5.9) N=15 | 12.0(7.6) | 18.2(4.0) |
| Atorvastatin (mg), mean (SD) | - | - | - | 40.0 (-) N=2 | 40.0(0) | - |
| Pravastatin (mg), mean (SD) | - | - | - | - | - | - |
| Fluvastatin (mg), mean (SD) | - | - | - | - | - | - |

**Table S2. Characteristics of de novo tretated patients with and without myopathy.**

^a^ Estimated GFR according to Cockcroft-Gault

^b^ Men>14 glasses/week =168 gram alcohol/week Women > 9glasses alcohol/week=108 gram alcohol/week

^c^ Exercise regularly= exercise at least twice weekly.

*p= 0.017
